# Deposition of Silicon-Based Stacked Layers for Flexible Encapsulation of Organic Light Emitting Diodes

**DOI:** 10.3390/nano9071053

**Published:** 2019-07-23

**Authors:** Chia-Hsun Hsu, Yang-Shih Lin, Hsin-Yu Wu, Xiao-Ying Zhang, Wan-Yu Wu, Shui-Yang Lien, Dong-Sing Wuu, Yeu-Long Jiang

**Affiliations:** 1 School of Opto-Electronic and Communication Engineering, Xiamen University of Technology, Xiamen 361024, China; 2 Department of Materials Science and Engineering, National Chung Hsing University, Taichung 40227, Taiwan; 3 Graduate Institute of Optoelectronic Engineering and Department of Electrical Engineering, National Chung Hsing University, Taichung 40227, Taiwan; 4 Department of Materials Science and Engineering, Da-Yeh University, Chunghwa 51591, Taiwan

**Keywords:** encapsulation, organic silicon, inorganic silicon, organic light emitting diode

## Abstract

In this study, inorganic silicon oxide (SiO*_x_*)/organic silicon (SiC*_x_*H*_y_*) stacked layers were deposited by a radio frequency inductively coupled plasma chemical vapor deposition system as a gas diffusion barrier for organic light-emitting diodes (OLEDs). The effects of thicknesses of SiO*_x_* and SiC*_x_*H*_y_* layers on the water vapor transmission rate (WVTR) and residual stress were investigated to evaluate the encapsulation capability. The experimental results showed that the lowest WVTR and residual stress were obtained when the thicknesses of SiO*_x_* and SiC*_x_*H*_y_* were 300 and 30 nm, respectively. Finally, different numbers of stacked pairs of SiO*_x_*/SiC*_x_*H*_y_* were applied to OLED encapsulation. The OLED encapsulated with the six-pair SiO*_x_*/SiC*_x_*H*_y_* exhibited a low turn-on voltage and low series resistance, and device lifetime increased from 7 h to more than 2000 h.

## 1. Introduction

Organic light-emitting diodes (OLEDs) have been attracting increasing attention recently because of their low production costs, fast response time, flexibility, and high efficiency. However, the organic and electrode materials in OLED devices are very sensitive to moisture and oxygen, making it important to develop encapsulation against moisture permeation. To solve these problems, the typical glass-lid encapsulation method has been used [1]. This encapsulation method is reliable and quite simple, but the brittle nature of glass means it cannot be applied to flexible devices. For flexible OLED devices, thin-film encapsulations are regarded as an alternative technology with immense prospects [2,3,4]. In particular, the organic–inorganic multilayer structure of thin-film encapsulation is a promising technology [5,6,7,8,9,10]. Several types of chemical vapor deposition (CVD) techniques are applied for the deposition of silicon-based thin films including low pressure CVD [11], plasma enhanced CVD [12], and inductively coupled plasma CVD (ICPCVD) [7,13,14], which all have certain characteristics. Among these, ICPCVD technology is promising and is widely used as a high-density plasma source. The high-density plasma enables the deposition of high-quality silicon-based thin film at lower temperatures. In addition, ICPCVD provides higher deposition rates due to higher dissociation efficiency and low plasma sheath potential near the chamber wall, reducing ion bombardment and enhancing uniformity [14]. In our previous works, properties of silicon oxide (SiO*_x_*) and organic silicon (SiC*_x_*H*_y_*) films prepared by ICPCVD were investigated [15,16]. Organic silicon usually refers to compounds containing silicon-carbon and carbon-hydrogen bonds [17,18].

In this paper, inorganic SiO*_x_* and organic SiC*_x_*H*_y_* stacked layers were prepared using ICPCVD as thin-film encapsulation for OLED devices. The thickness of the SiO*_x_* layers was varied in order to reduce the water vapor transmission rate (WVTR). Additionally, to reinforce the effects provided by the SiO*_x_* layer, we introduced SiC*_x_*H*_y_* layers to offset the residual stress of the SiO*_x_* layer. The properties of WVTR and residual stress for different pairs of SiO*_x_*/SiC*_x_*H*_y_* stacked layers were investigated. Finally, the six-pair SiO*_x_*/SiC*_x_*H*_y_* stacked layer was applied to OLED encapsulation and showed excellent current–voltage–luminance characteristics and stable lifetime performance.

## 2. Materials and Methods

The SiO*_x_* and SiC*_x_*H*_y_* layers were prepared on polyethylene terephthalate (PET) substrates (5 × 5 cm^2^) by a 13.56 MHz radio frequency ICPCVD system. A gas mixture of oxygen (O_2_) and tetramethylsilane (TMS) was used for deposition of SiO*_x_*, while argon (Ar) and TMS were used to deposit SiC*_x_*H*_y_*. The detailed deposition parameters of SiO*_x_* and SiC*_x_*H*_y_* thin films are summarized in Table 1. The WVTR of the films was measured by a WVTR permeation instrument (Permatran-WR Model 3/61, Mocon Inc., Minneapolis, MN, USA) under the conditions of 40 °C and 100% relative humidity (RH) [19,20]. The residual stress of the SiO*_x_* and SiO*_x_*/SiC*_x_*H*_y_* layers was measured on silicon substrates by a laser profilometer (FLX-2320, Tencor, Milpitas, CA, USA) using Stoney’s equation [21]:

(1) $$\sigma =\frac{Eh^{2}}{(1-\upsilon )6Rt}$$

(2) $$R=\frac{R_{1}R_{2}}{R_{1}-R_{2}}$$

where *σ* is the in-plane stress component in the film, *E* is Young’s modulus of the substrate, *h* denotes the thickness of the substrate, *ν* is Poisson’s ratio of the substrate, *t* is the thickness of the film, and *R*_1_ and *R*_2_ are the radii of curvature of the substrate before and after film deposition, respectively. The thin-film encapsulation of OLED devices was carried out using the SiO*_x_* (300 nm)/SiC*_x_*H*_y_* (30 nm) stacked layers. The pair number of the stacked layers was varied from 0 to 8. The current density–voltage (J–V) and luminance–voltage (L–V) characteristics were recorded simultaneously with the electroluminescence spectra by combining the PR650 spectrometer (Photo Research Inc., Chatsworth, CA, USA). The lifetime of the OLED device, defined as the time required for the luminance to degrade to 0%, was measured using an OLED lifetime test system (model 58131, Chroma ATE Inc., Taoyuan, Taiwan) under the conditions of 22 °C temperature and 70% RH. Finally, the cross-section microstructure of the OLED device was examined using transmission electron microscopy (TEM, Philips, Bend, OR, USA) and scanning electron microscopy (SEM, JEOL USA Inc., Peabody, MA, USA).

## 3. Results

**Figure 1:**
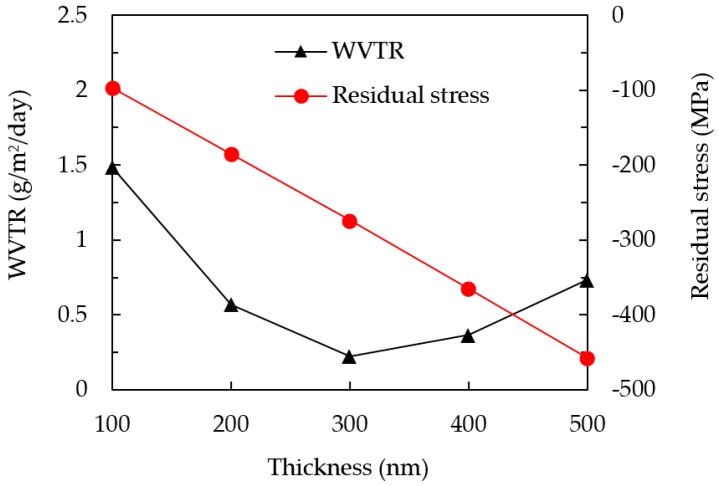
Water vapor transmission rate (WVTR) and residual stress of the SiO*_x_* films with different thicknesses.

It is necessary for SiO*_x_* to have a thickness of a few micrometers for it to be used as an encapsulation layer. However, a thick SiO*_x_* single layer is usually accompanied by micro-cracks in the films due to the large residual stress. The main idea of this work was to prepare multiple SiO*_x_*/SiC*_x_*H*_y_* layers to encapsulate OLEDs on flexible substrates. The critical values of the thickness and residual stress for the SiO*_x_* single layer need to be determined, and then the SiO*_x_* can be stacked with a SiC*_x_*H*_y_* layer of an appropriate thickness to balance the residual stress. Thus, by repeating the SiO*_x_*/SiC*_x_*H*_y_* structure, the total thickness of the SiO*_x_* layer can be significantly increased. Figure 1 shows the WVTR at 40 °C/100% RH and the residual stress of the SiO*_x_* layers with different thicknesses, ranging from 100 to 500 nm. It can be seen that the SiO*_x_* with a thickness of 300 nm has the lowest WVTR value of 0.23 g/m^2^/day. The residual stress of the SiO*_x_* layers decreases from −97 to −458 Mpa as the thickness increases from 100 to 500 nm. The negative value sign represents the compressive stress, while the positive value sign is assigned to the tensile stress. The compressive stress increases with the increasing SiO*_x_* thickness. Compared to the 300 nm-thickness film, the SiO*_x_* films with thicknesses of 400 and 500 nm have an increased WVTR, due to their high compressive stresses leading to some micro-cracking in the SiO*_x_*. A similar relation between crack morphology and permeation of moisture and oxygen on PET has been reported earlier [22,23]. Therefore, a layer with tensile stress (i.e., SiC*_x_*H*_y_*) was expected to help compensate for the compressive stress of the SiO*_x_* layer.

**Figure 2:**
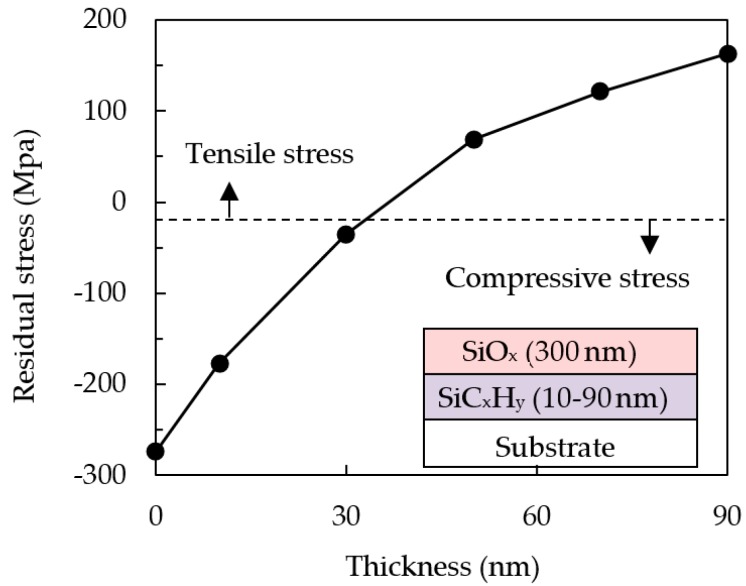
Residual stress of the SiO*_x_*/SiC*_x_*H*_y_* films with different SiC*_x_*H*_y_* thicknesses.

Figure 2 shows the residual stress of SiO*_x_*/SiC*_x_*H*_y_* stacked layers at different SiC*_x_*H*_y_* thicknesses (10–90 nm) while the SiO*_x_* thickness is kept at 300 nm. The SiC*_x_*H*_y_* layer is designed to be placed beneath the SiO*_x_* layer, as the SiC*_x_*H_y_ layer can improve the adhesion of the SiO*_x_* to the substrate. It is found that the SiO*_x_*/SiC*_x_*H*_y_* stacked layers change from compressive stress to tensile stress, and the 30 nm-thickness is the most suitable since the total residual stress is about −31 MPa, the closest to zero. Thus, the overall thickness of SiO*_x_* can easily be increased to provide improved moisture resistivity while maintaining a low residual stress by increasing the stacked pair number of SiO*_x_* (300 nm)/SiC*_x_*H*_y_* (30 nm). Although not shown here, a WVTR at 10^−6^ g/m^2^/day level can be reached when the stacked pair number is equal to or larger than six. For examples, the WVTR values of the six-pair and eight-pair SiO*_x_*/SiC*_x_*H*_y_* are 8 × 10^−6^ and 6 × 10^−6^ g/m^2^/day, respectively. These values satisfy the requirement of OLED encapsulation, which is suggested to be at least at a level of 10^−6^.

**Figure 3:**
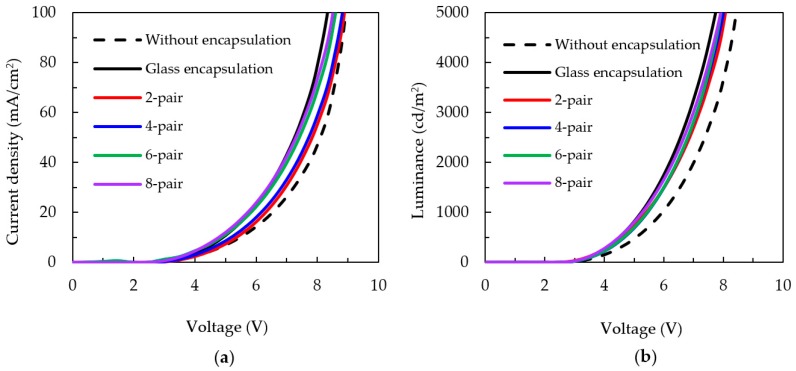
(**a**) Current density–voltage and (**b**) luminance–voltage characteristics of organic light-emitting diodes (OLED) devices encapsulated by different pair numbers of SiO*_x_*/SiC*_x_*H*_y_* stacks.

Figure 3a shows the J–V characteristics of OLED devices without encapsulation, with glass encapsulation, and with different numbers of pairs of SiO*_x_*/SiC*_x_*H*_y_* encapsulation. The series resistance (*R*_s_) for the devices is sensitive to the encapsulation, as moisture diffused into the devices may cause deterioration in the properties of the electrode and the organic layer of the devices. *R*_s_ can be estimated from the reciprocal slope at the high voltage region of the J–V curves. The values of *R*_s_ were 3.11, 2.32, 1.95, 1.71, 1.58, and 1.11 for the device without encapsulation and those with two-pair, four-pair, six-pair, eight-pair, and glass encapsulation, respectively. The turn-on voltage, defined as the voltage when the luminance reaches 1 cd/m^2^, showed a similar trend to *R*_s_. The turn-on voltages were about 2.53, 2.51, 2.50, 2.49, 2.47, and 2.14 V for the device without encapsulation and those with two-pair, four-pair, six-pair, eight-pair, and glass encapsulation, respectively. The lower the turn-on voltage, the less power the OLED device consumes. This is also reflected in the L–V characteristics shown in Figure 3b. A device with a lower turn-on voltage requires less power input to reach a certain luminance. Therefore, for thin-film encapsulation, OLEDs with the six- and eight-pair SiO*_x_*/SiC*_x_*H*_y_* can be expected to have high luminous efficiency and low power consumption.

**Figure 4:**
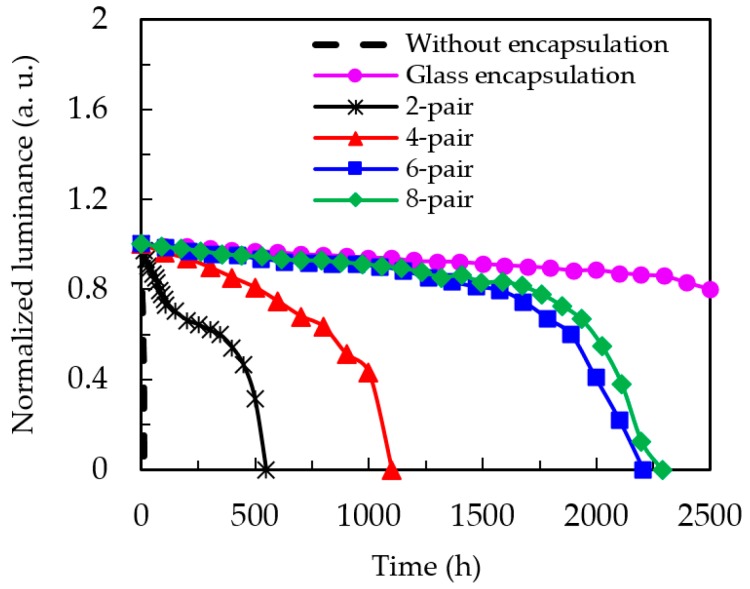
Normalized luminance of the OLED devices without encapsulation, with glass encapsulation, and with different numbers of pairs of SiO*_x_*/SiC*_x_*H*_y_* encapsulation as a function of operation time.

Figure 4 shows the lifetime testing results for OLED devices without encapsulation, with glass encapsulation, and with different numbers of pairs of SiO*_x_*/SiC*_x_*H*_y_* encapsulation. The reduction of the luminance for the devices was recorded to evaluate the lifetime of the OLEDs and the performance of the encapsulation barriers. The tested devices were placed in a constant environment at 22 °C and 70% RH. In the OLED device with the standard glass-lid encapsulation the luminance decreased slowly, and the device lifetime was over 2500 h. The OLED devices with six-pair and eight-pair encapsulations exhibited lifetimes around 2000 h. The reduced lifetime for thin-film encapsulation samples may be ascribed to the thin-film deposition process that leads OLED devices to inevitably have contact with water-vapor residing in the ICPCVD chamber. Due to the needs for reductions of the weight and thickness of optoelectronic devices, the substitution of thin-film encapsulation for glass encapsulation is indispensable. Considering the fabrication time and performance, the six-pair SiO*_x_*/SiC*_x_*H*_y_* layer demonstrated great potential for OLED encapsulation.

**Figure 5:**
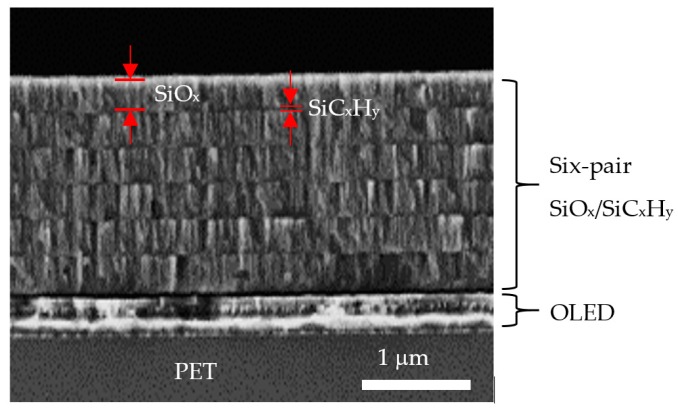
Cross-sectional scanning electron microscope image of the OLED device with six-pair SiO*_x_*/SiC*_x_*H*_y_* encapsulation.

The SEM cross-sectional image of the OLED device with six-pair SiO*_x_*/SiC*_x_*H*_y_* stacked layer encapsulation is shown in Figure 5. It can be clearly seen that the SiO*_x_* (300 nm)/SiC*_x_*H*_y_* (30 nm) single-pair and the six-pair with a total thickness of 1980 nm are included. Additionally, we note that the interfaces are smooth without observable cracks or voids, and the encapsulation layers are well adhered to the bottom of the OLED device. The greater thickness, smooth interfaces, and absence of cracks of the encapsulation layers can therefore provide remarkable water-vapor resistivity to protect OLEDs from the outer environment.

**Figure 6:**
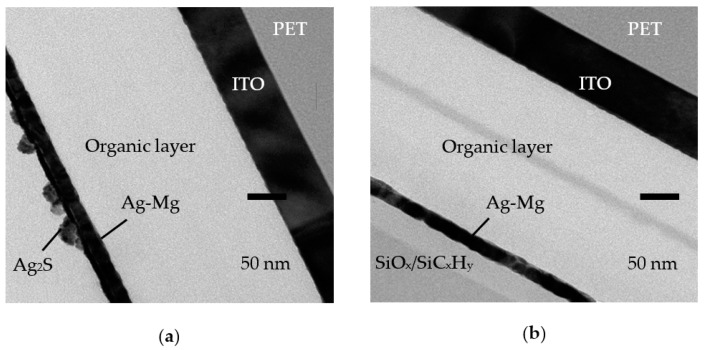
Cross-sectional TEM images of the OLED devices (**a**) without encapsulation, and (**b**) with six-pair SiO*_x_*/SiC*_x_*H*_y_* encapsulation.

Figure 6 shows the cross-sectional TEM images for the OLED devices without and with the six-pair encapsulation barrier with the operation time of 30 h at 22 °C and 70% RH. In Figure 6a, the device without encapsulation shows the corroded Ag-Mg electrode with Ag_2_S formation, which may be formed on the electrode after exposure to a humid environment [24,25]. This will further offer a path for moisture and oxygen to diffuse into the organic layer of OLEDs to eventually form spots or dead areas. In contrast, the six-pair SiO*_x_*/SiC*_x_*H*_y_* encapsulation for the OLED device does not show corrosion of the electrode. There is no observable Ag_2_S or defect on Ag-Mg, as shown in Figure 6b.

**Figure 7:**
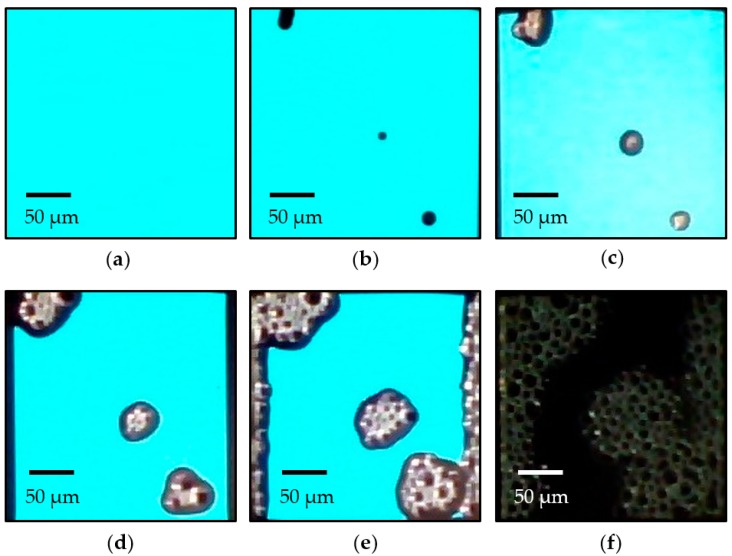
Photographs of the OLED devices with six-pair encapsulation for (**a**) 1200, (**b**) 1400, (**c**) 1600, (**d**) 1800, (**e**) 2000, and (**f**) 2200 h.

Figure 7 shows the photographic images of the OLED device with six-pair SiO*_x_*/SiC*_x_*H*_y_* encapsulation recorded at an interval of 200 h under 22 °C and 70% RH. No spots can be observed on the device before 1200 h, and a representative image of the OLED devices is shown in Figure 7a. After then, spots occur and their area ratio was calculated using TracePro computing software (version 6.0, Bethesda, Rockville, MD, USA). For an example, the OLED shows a spot area of about 2.9% at the operation time of 1400 h, as shown in Figure 7b. Note that these spots are still very small and hardly visible. The area of the spot drastically increases at 1600 h (Figure 7c). At 1800 and 2000 h, the significant dark area on the left and right sides might be evidence that the moisture can enter from the sides of the encapsulated devices. This may not occur in the case of glass-lid encapsulation, which has glue on the sides of the glass to resist moisture diffusion. As a consequence, the lifetime of the six-pair encapsulated OLED is shorter than that of the glass encapsulated device, as shown in Figure 4. At around 2200 h, the OLED device turns off (Figure 7f).

## 4. Conclusions

The silicon-based SiO*_x_* and SiC*_x_*H*_y_* layers were prepared on PET using the ICP-CVD system. In order to offset the residual stress, the thicknesses of SiO*_x_* and SiC*_x_*H*_y_* layers were optimized. The residual stress value was closest to zero for the 300 nm SiO*_x_* stacked with 30 nm SiC*_x_*H*_y_*. The performance of OLED devices is dependent on the thin-film encapsulation, especially the quality of the SiO*_x_*/SiC*_x_*H*_y_* stacked layers. The six-pair SiO*_x_*/SiC*_x_*H*_y_* stacked layer encapsulation for OLED device with low *R*_s_, low turn-on voltage of 2.49 V, and a lifetime of over 2000 h showed excellent protection from ambient moisture. These results suggest that six-pair SiO*_x_*/SiC*_x_*H*_y_* stacked layer thin-film encapsulation has great potential for OLED device encapsulation and provides an alternative to traditional glass-lid encapsulation.

## Figures and Tables

**Table 1:** Deposition parameters for SiO*_x_* and SiC*_x_*H*_y_* thin films.

Parameter	SiO*_x_*	SiC*_x_*H*_y_*
Power (W)	600	400
Pressure (mTorr)	5	5
TMS flow (sccm)	60	10
O_2_ flow (sccm)	2	—
Ar flow (sccm)	—	40
Temperature (°C)	90	90
Thickness (nm)	100–500	10–90
